# Precise stacking of decellularized extracellular matrix based 3D cell-laden constructs by a 3D cell printing system equipped with heating modules

**DOI:** 10.1038/s41598-017-09201-5

**Published:** 2017-08-17

**Authors:** Geunseon Ahn, Kyung-Hyun Min, Changhwan Kim, Jeong-Seok Lee, Donggu Kang, Joo-Yun Won, Dong-Woo Cho, Jun-Young Kim, Songwan Jin, Won-Soo Yun, Jin-Hyung Shim

**Affiliations:** 1 Research Institute, T&R Biofab Co. Ltd., 237 Sangidaehak-Ro, Siheung, 15073 Republic of Korea; 2 Department of Mechanical Engineering, Korea Polytechnic University, 237 Sangidaehak-Ro, Siheung, 15073 Republic of Korea; 3 Department of Mechanical System Engineering, Korea Polytechnic University, 237 Sangidaehak-Ro, Siheung, 15073 Republic of Korea; 4 Department of Mechanical Engineering, Pohang University of Science and Technology (POSTECH), Pohang, 37673 Republic of Korea; 5 Department of Orthopedic Surgery, Kyungpook National University Hospital, Daegu, 41944 Republic of Korea

## Abstract

Three-dimensional (3D) cell printing systems allow the controlled and precise deposition of multiple cells in 3D constructs. Hydrogel materials have been used extensively as printable bioinks owing to their ability to safely encapsulate living cells. However, hydrogel-based bioinks have drawbacks for cell printing, e.g. inappropriate crosslinking and liquid-like rheological properties, which hinder precise 3D shaping. Therefore, in this study, we investigated the influence of various factors (e.g. bioink concentration, viscosity, and extent of crosslinking) on cell printing and established a new 3D cell printing system equipped with heating modules for the precise stacking of decellularized extracellular matrix (dECM)-based 3D cell-laden constructs. Because the pH-adjusted bioink isolated from native tissue is safely gelled at 37 °C, our heating system facilitated the precise stacking of dECM bioinks by enabling simultaneous gelation during printing. We observed greater printability compared with that of a non-heating system. These results were confirmed by mechanical testing and 3D construct stacking analyses. We also confirmed that our heating system did not elicit negative effects, such as cell death, in the printed cells. Conclusively, these results hold promise for the application of 3D bioprinting to tissue engineering and drug development.

## Introduction

Three-dimensional (3D) cell printing systems have been developed to generate artificial tissues or organs in the field of tissue engineering^1–7^. These systems are powerful tools for the controlled deposition of biomaterials and cells to form mature tissues or organs. Mimicking the forms and functions of native tissues and organs are major challenges in the fabrication of artificial tissue and organ constructs via 3D cell printing systems^6, 8–16^.

Hydrogel materials (e.g. collagen, gelatin, and alginate) are widely used as bioinks in 3D cell printing systems owing to their capacity for cell encapsulation and printability^3, 6, 17–22^. In particular, collagen hydrogels are commonly utilized for the regeneration of specific tissues, such as the skin^3, 21^, vasculature^23, 24^, bone^15, 19^, liver^25^, and nerves^26^ because collagen is the most abundant protein-based natural polymer in mammalian tissues and is a main component of the native extracellular matrix (ECM), capable of providing a favourable microenvironment^6, 12, 15^.

Hydrogel crosslinking is critical for the maintenance of the original 3D structure, which is necessary to provide a microenvironment with proper mechanical properties for cellular activities^27, 28^. Various crosslinking processes have been developed for collagen-based hydrogels, including thermal^29^, chemical^30, 31^, and photo-crosslinking^28, 32^. However, harmful crosslinking reagents for hydrogel gelation, such as glutaraldehyde, EDC (1-ethyl-3-(3-dimethylaminopropyl) carbodiimide), and photo-initiators, cause poor biocompatibility and high cytotoxicity owing to the residues produced after crosslinking^33–35^. In contrast, crosslinking via thermal heating is simple and safe for cellular activities. Despite the high biocompatibility, the gelation time for thermal crosslinking is longer than those for other processes.

The most important characteristic of 3D cell printing is the capacity for the deposition of a bioink with live cells at the desired position by a layer-by-layer process to generate a 3D culture environment^17, 20, 22, 28^. For the successful generation of artificial tissues or organs using 3D cell printing technology, an adequate 3D bioprinter, printing process, and bioink are needed^19, 20, 22, 28, 36, 37^. In particular, the rheological properties of bioinks, such as viscosity and printability, are critical. In cases of high viscosity, printability, including shape fidelity, is generally excellent, but cell viability is low. Thus, there is a tradeoff between cell viability and printability. In contrast, bioinks with low viscosity have liquid-like properties, resulting in the collapse of 3D-printed constructs in a layer-by-layer process^19, 20, 22, 36–38^. As cellular activities, such as proliferation and differentiation, in bioinks are critical to the maturation of artificial tissues or organs^20^, suitable rheological properties (i.e. low viscosity) directly related to the cellular microenvironment should be considered during the 3D cell printing process. In addition, the printing accuracy and structural integrity of 3D-printed constructs are also important for the successful fabrication of 3D tissue analogues during long-term culture^20, 22, 36^.

Herein, we developed a 3D cell printing system with heating modules for the simultaneous crosslinking of dECM bioink. The purpose of this study was to establish a 3D cell printing process that includes a bioink with suitable rheological properties and a safe and rapid crosslinking procedure during printing, without any additional steps. We demonstrated that a 3D cell printing system equipped with a heating system dramatically improved the printing fidelity of 3D constructs consisting of dECM bioink with no cytotoxicity.

## Results

### Preparation and characterization of bioinks

**Figure 1:**
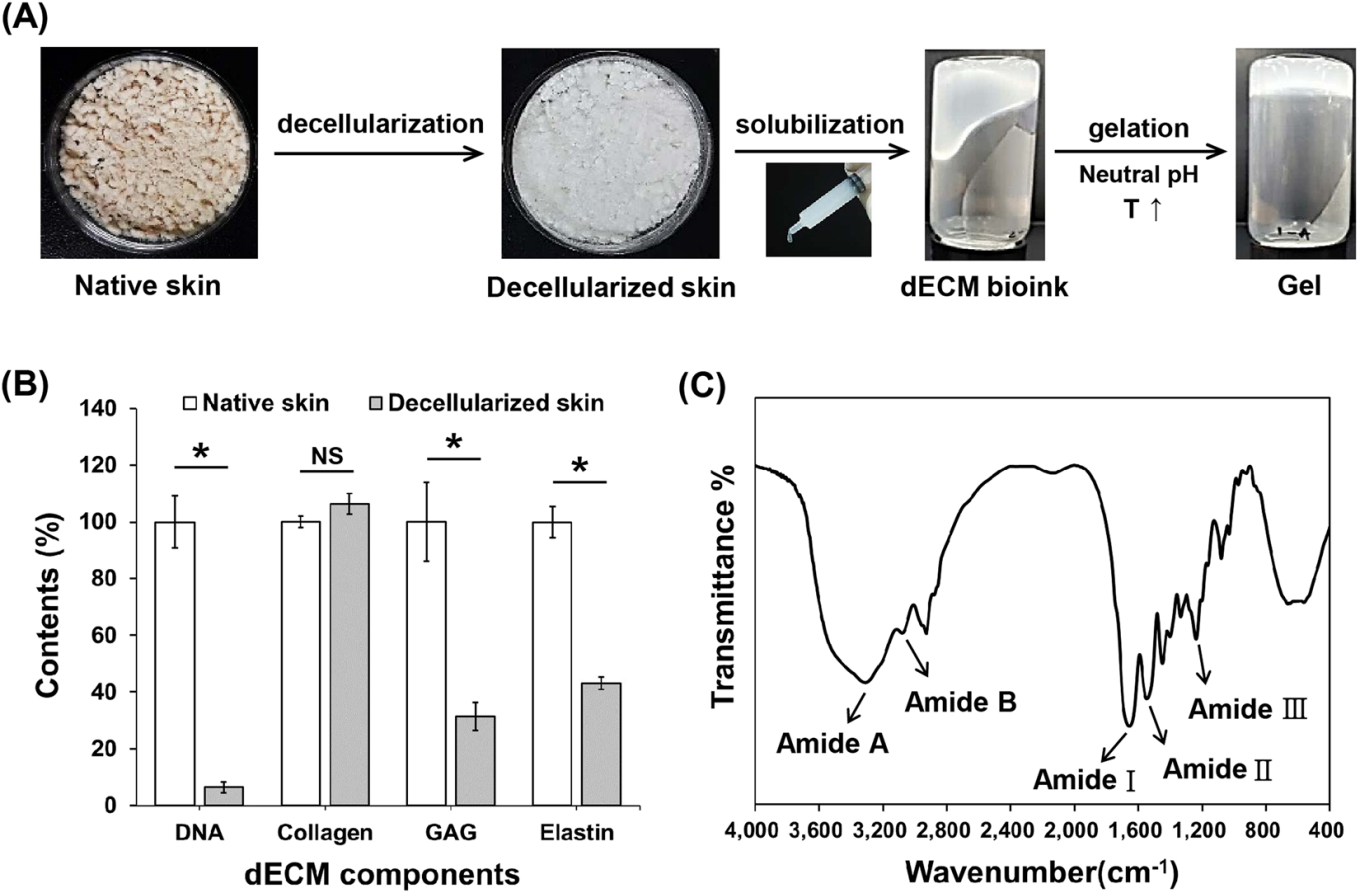
Characteristics of skin-derived dECM. (**A**) Process for extraction of skin-derived collagen bioink and gelation behaviour. (**B**) Quantitative analysis of dECM components. (*p < 0.001) (**C**) FTIR spectra of the skin-derived dECM.

Skin-derived dECM was produced by decellularization and solubilization processes from native porcine-derived skin tissues (Fig. 1(A)). As shown in Fig. 1(B), most DNA from native skin tissues was successfully removed. The DNA quantity in decellularized skin was significantly lower than that of native skin (*p* < 0.001). Among the other dECM components, collagen content was slightly increased and glycosaminoglycans (GAGs) and elastin contents were notably decreased (*p* < 0.001) during the decellularization process.

FTIR spectra of dECM are shown in Fig. 1(C); these results confirmed that tissue-derived collagen was mainly comprised of collagen proteins. The dECM showed typical amide bands at 1651, 1543, and 1236 cm^−1^, indicating C = O stretching (Amide I), N-H deformation (Amide II), and N-deformation (Amide III), respectively. Additionally, Amide A (indicating the stretching N-H bonds) and Amide B (indicating the stretching vibrations of N-H) bands were observed at 3,300 and 3,070 cm^−1^.

### Effects of bioink concentrations on cell viability

**Figure 2:**
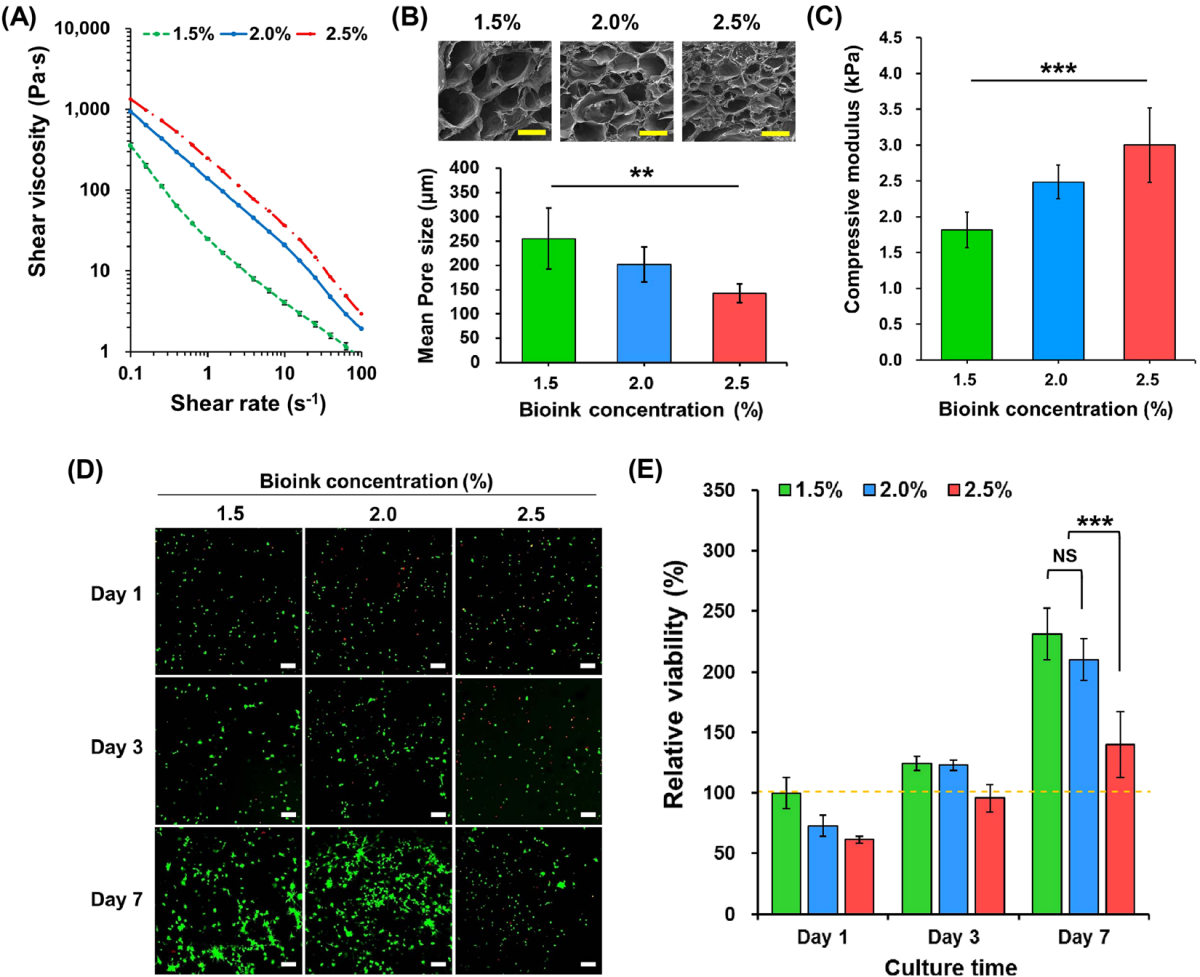
Analysis of dECM bioink with respect to physical and biological properties. (**A**) Shear viscosity, (**B**) SEM images and mean pore sizes (yellow scale bar: 500 μm; **p < 0.005), and (**C**) compressive moduli of bioinks with various concentrations (***p < 0.05). (**D**) Live/dead assay results of NIH3T3 cells within bioinks with various concentrations (1.5%, 2.0%, and 2.5%) for 7 days (white scale bar: 100 μm). (**E**) Relative viability (%) results. (***p < 0.05, NS = no significant difference).

The viscosity of the bioink with respect to the concentration is summarised in Fig. 2(A). Higher concentrations of bioink (2.5%) were associated with a higher viscosity; this can be attributed to the relative increase in polymer chains in the bioink^39^. Furthermore, different concentrations of gelled bioinks exhibited different pore sizes. As shown in Fig. 2(B), the mean pore size of 1.5% gelled bioinks (approximately 250 μm) was significantly higher than those of the other gelled bioinks (2.0%: approximately 200 μm; 2.5%: approximately 140 μm; *p* < 0.005). The compressive mechanical properties of the gelled bioinks were consistent with the viscosity behaviours. The compressive moduli gradually increased as the bioink concentration increased (Fig. 2(C); *p* < 0.05). To confirm the suitable bioink concentration for cellular activity, we performed cell viability (using NIH3T3 cells) tests with bioinks at various concentrations. As shown in Fig. 2(D), when a relatively high bioink concentration (2.5%) was used, relative cell viability when the value of 1.5% bioink at day 1 was taken as 100% was low during a 7-day period, whereas relatively lower concentrations (1.5% and 2.0%) of bioinks resulted in a high cell viability (Fig. 2(E)).

### Effects of bioink concentration on printing parameters in 2D patterning

**Figure 3:**
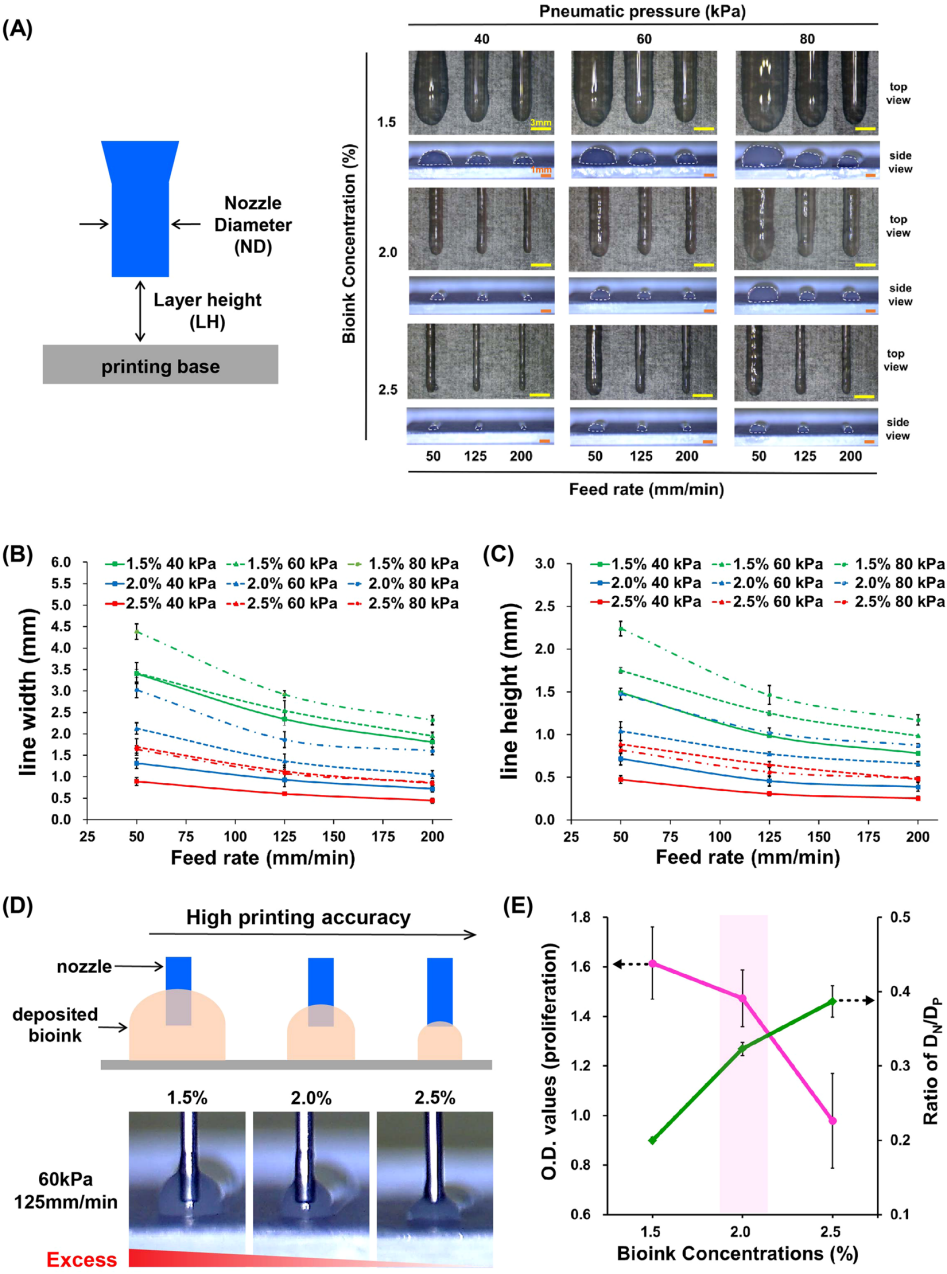
2D patterning analysis. (**A**) Schematic diagram of printing settings and 2D patterning results depending on bioink concentrations and printing parameters. Quantitative analysis of the (**B**) width and (**C**) height of the printed straight lines. (**D**) Definition of printing accuracy and its representative optical images. (**E**) The relationship between cell proliferation and printing precision (ratio of D_N_/D_P_).

We further examined the effects of bioink concentration, pneumatic pressure, and feed rate on 2D patterning with 1.5, 2.0, and 2.5% bioink. Figure 3(A) shows the top and side view images of the dispensed bioinks for various printing conditions. Notably, the dispensing volume for bioinks with a relatively low concentration was higher than that for bioinks with a relatively high concentration under identical printing conditions. In terms of printing parameters, a high pneumatic pressure and low feed rate led to a higher dispensing volume than those observed for the opposite conditions. These conditions influenced the characteristics of line shapes, such as the width and height of lines (Fig. 3(B,C)). In 2D patterning, the nozzle diameter and the predetermined layer height used in this study were both 0.25 mm. However, the range of printed line widths was 0.45–4.3 mm, and the range of line heights was 0.25–2.24 mm. The dispensed bioink appeared to have a spreadable nature because the bioink did not immediately gel. As a result, the line width of the bioink dispensed with the 0.25 mm nozzle was approximately 1.8- to 17.2-times greater than the nozzle diameter. Furthermore, the line height was approximately 1.0- to 9.0-times greater than the nozzle diameter. For semi-quantification of 2D patterning, we used the ratio (D_N_/D_P_) of the nozzle diameter (D_N_) and the converted diameter of the dispensed lines (D_P_) based on the cross-sectional area of the lines, as indicative of printing precision. The D_P_ was the diameter calculated by the area of the dispensed lines, which was a non-spherical shape. The ratio is higher when the lines of a predetermined design are printed more precisely by bioinks under the same printing conditions. The cross-sectional area was calculated from the side view images in Fig. 3(A). Figure 3(D) shows the printing tendencies according to the extent of dispensing. A high feed rate yielded a thinner line that could result in a break, despite an enhanced PA^10^. Under the same printing conditions, the dispensing volume of low-concentration bioink was excessive, whereas that of high-concentration bioink was moderate under the predetermined settings. Based on these analyses, we concluded that the most suitable printing conditions were as follows: a bioink concentration of 2.0%, pneumatic pressure of 60 kPa, and feed rate of 125 mm/min. The relationship between cell viability (proliferation) for various concentrations of bioinks and printing precision is shown in Fig. 3(E).

### Printing of 3D constructs

**Figure 4:**
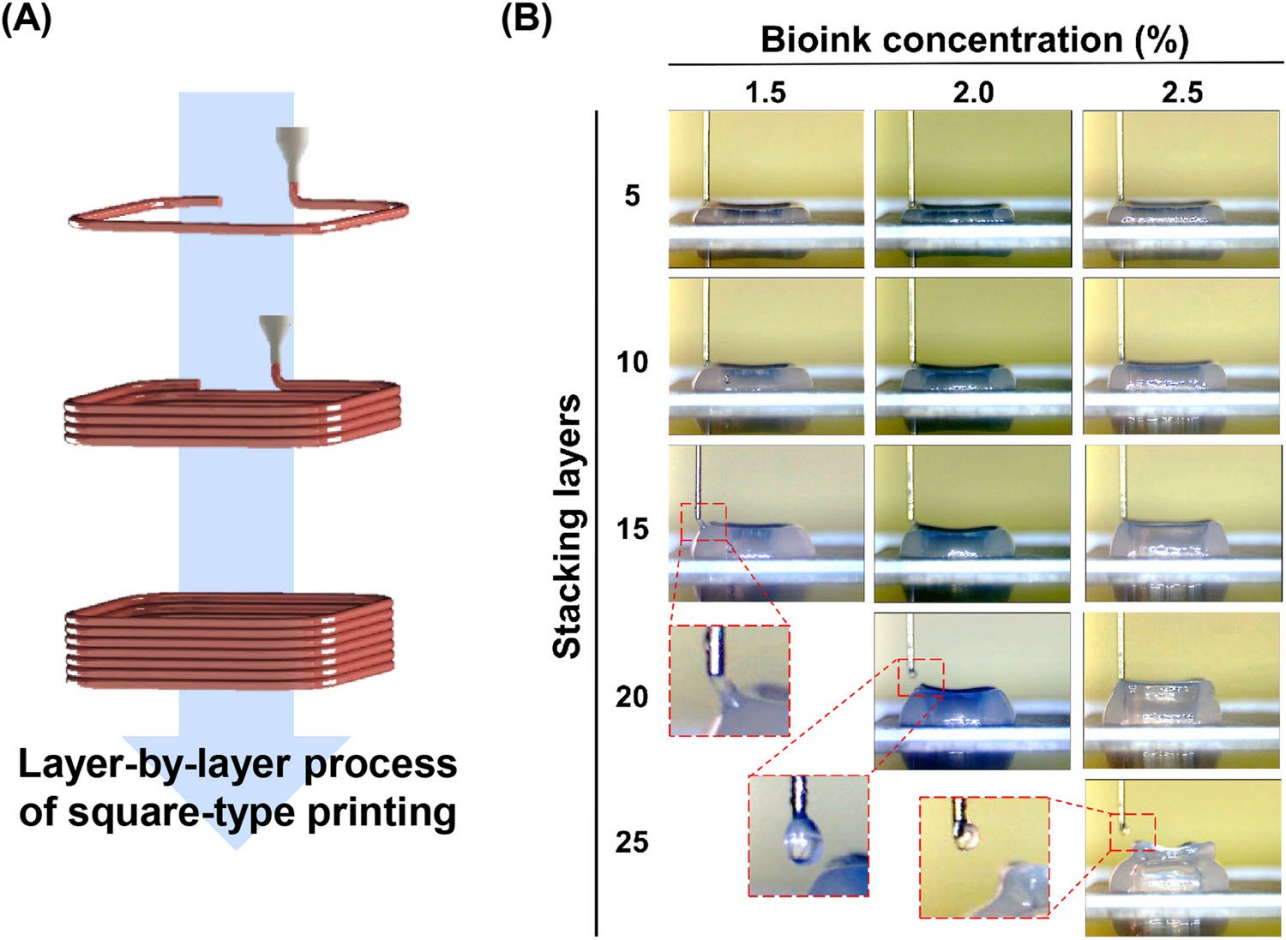
3D stacking process. (**A**) Schematic illustration of layer-by-layer process for 3D printing, and (**B**) 3D stacking results of bioinks with different concentrations.

**Figure 5:**
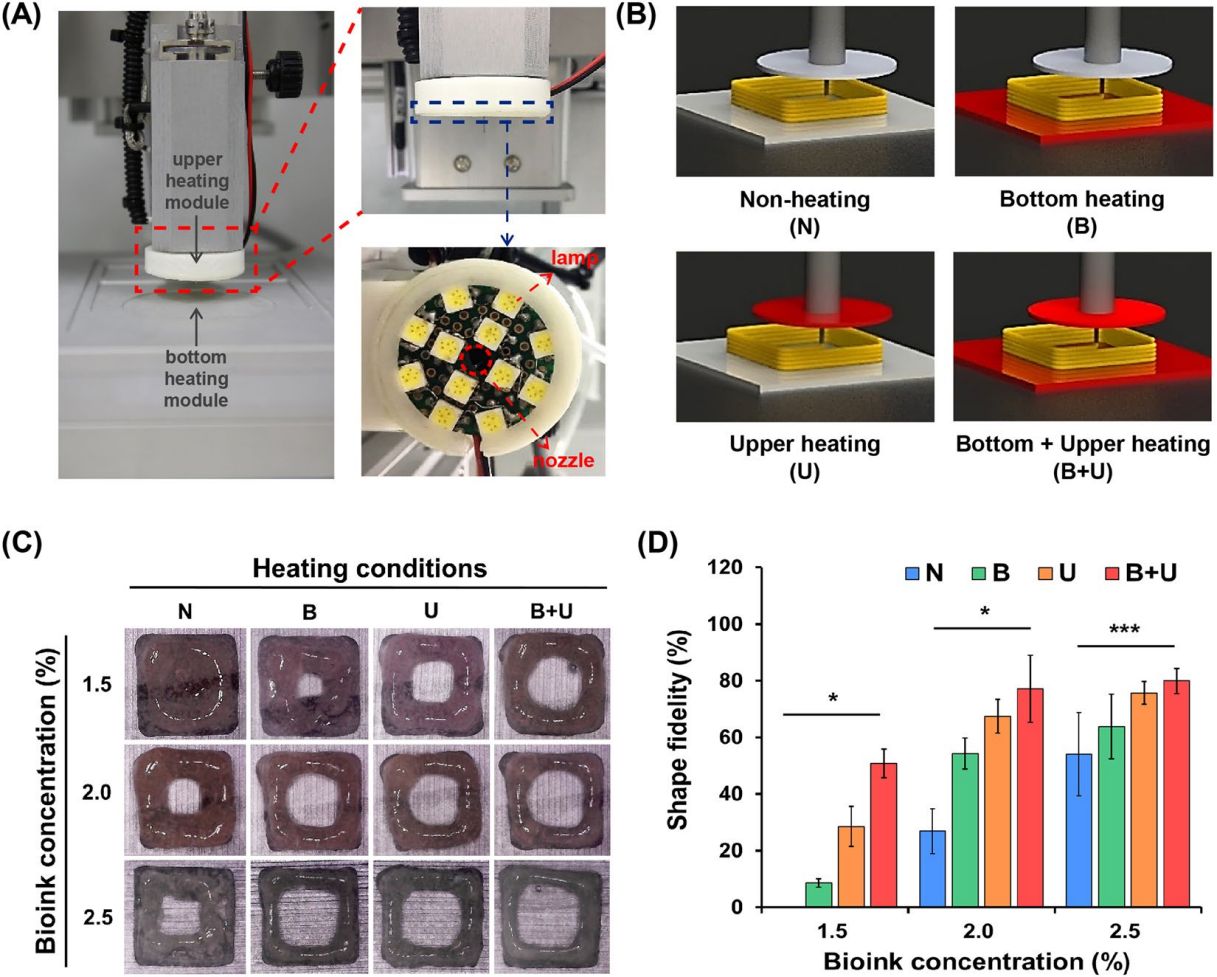
Heating systems and their efficacy. (**A**) Photographs of the upper and bottom heating modules installed in 3D printing equipment. (**B**) Conceptual diagram of non-heating and heating conditions. (**C**) Representative optical images of 3D-printed hollow-type squares, and (**D**) Quantitative analysis of shape fidelity under different bioink concentrations and various heating conditions. (*p < 0.001, ***p < 0.05).

We confirmed the limitations of bioink stacked along the *z*-direction depending on the bioink concentration. The process is shown in a schematic diagram in Fig. 4(A and B) shows the 3D stacking results for different bioink concentrations. Without the simultaneous gelation of bioinks, the collapse phenomenon was expected owing to the liquid-like properties of the bioink and the effect of gravity^36^. The number of stacking layers obtained with a relatively high concentration (2.5%) of bioink was higher than that obtained for other concentrations. Additionally, there was a considerable gap between the end of the nozzle and the surface of the final layer owing to the collapse phenomenon, as shown in the lower panels in Fig. 4(B). Thus, these results showed that higher concentrations of bioink resulted in less collapse owing to the increased viscoelastic properties, providing a self-supporting ability. Although the shape-making ability of high-concentration bioink was excellent, the microenvironment for live cells was inadequate, similar to the cell viability results described above (Fig. 3(E)). Therefore, we applied our new 3D cell printing system equipped with heating modules for the simultaneous gelation of dispensed bioinks. Upper and lower heating modules were installed in our 3D printing system, as shown in Fig. 5(A). Figure 5(B) shows a schematic illustration of various heating conditions (bottom [B], upper [U], and bottom plus upper [B + U] heating) used in this study. A predesigned 3D hollow-type square with an empty region at the centre was printed under different heating conditions using various bioink concentrations to evaluate the heating effect and stacking ability (Fig. 5(C)). The heating system improved printability in this experiment, especially in the B + U condition. For a quantitative analysis of 3D printability, a shape fidelity (SF) parameter was calculated using the following equation:

1 $${\rm{Shape}}\,{\rm{fedelity}}\,({\rm{SF}})\,( \% )=\frac{{{\rm{A}}}_{{\rm{RE}}}}{{{\rm{A}}}_{{\rm{TE}}}}\,\times 100$$

where A_RE_ is the area of real empty space and A_TE_ is the area of theoretical empty space. The SF parameter represents the ratio between A_RE_ and A_TE_. A high SF corresponds to a high printability. Regardless of the bioink concentration, the heating system enhanced the SF. Notably, the heating effect in terms of SF was significantly greater when the bioink concentration was 1.5% or 2.0% than when the bioink concentration was 2.5% (Fig. 5(D)). Based on the cell viability and 3D stacking results according to the bioink concentrations, we determined that 2% bioink is suitable for printing.

**Figure 6:**
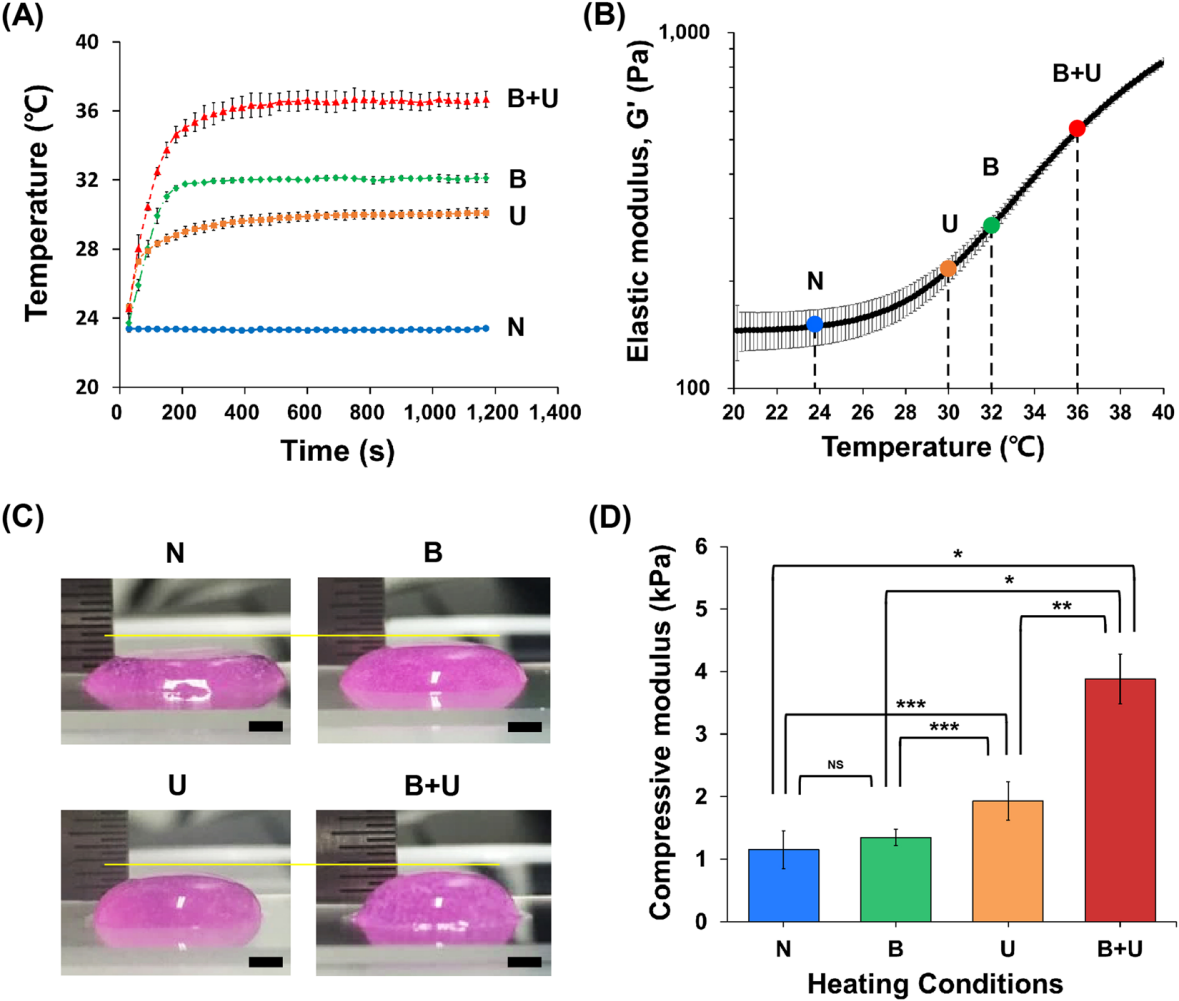
Efficacy of heating systems. (**A**) Results of saturation temperature measurement for each condition. (**B**) Temperature sweep oscillatory tests with a temperature ramp of 5°C/min from 20 °C to 40 °C. (**C**) 3D stacking results of bioinks with different heating condition. (**D**) Compressive moduli of the printed structures under each heating condition. (*p < 0.001, **p < 0.005, ***p < 0.05).

For an accurate analysis of the effects of heating systems on the 3D integration of the printed structures, the temperatures of each heating module were measured with thermocouples at a constant position (8 mm from the printing plate). The saturated temperatures of all heating conditions reached a steady-state after roughly 600 s and were approximately 23 °C, 30 °C, 32 °C, and 36 °C for N, B, U, and B + U, respectively (Fig. 6(A)). To understand the heating effect in detail, we observed changes in physical properties, such as rheology and compression characteristics, during heating. Figure 6(B) shows the changes in the elastic modulus (G′) of 2% bioink for various temperatures corresponding to each heating condition. As the temperature increased, the elastic modulus of the bioink increased. It indicated that the bioink was gelled in the heating condition. There were no major changes in G′ values under N conditions (corresponding to 23 °C); however, the G′ values under B, U, and B + U conditions increased gradually by partially gelled bioinks. Furthermore, the compression test verified that the heating conditions in actual printing changed the mechanical properties, i.e. the compressive modulus, of the printed structures with a diameter of 12 mm and height of 3 mm. Figure 6(C) shows the printed structures for the compression test. The printing results differed substantially among heating conditions. With respect to height, the structure printed under B + U was the least collapse. As shown in Fig. 6(D), heating during printing increased the compressive moduli of the printed structures due to the gelation of the printed structures. In particular, the compressive modulus in the B + U condition was significantly higher than those of N (*p* < 0.001), B (*p* < 0.001), and U (*p* < 0.005).

**Figure 7:**
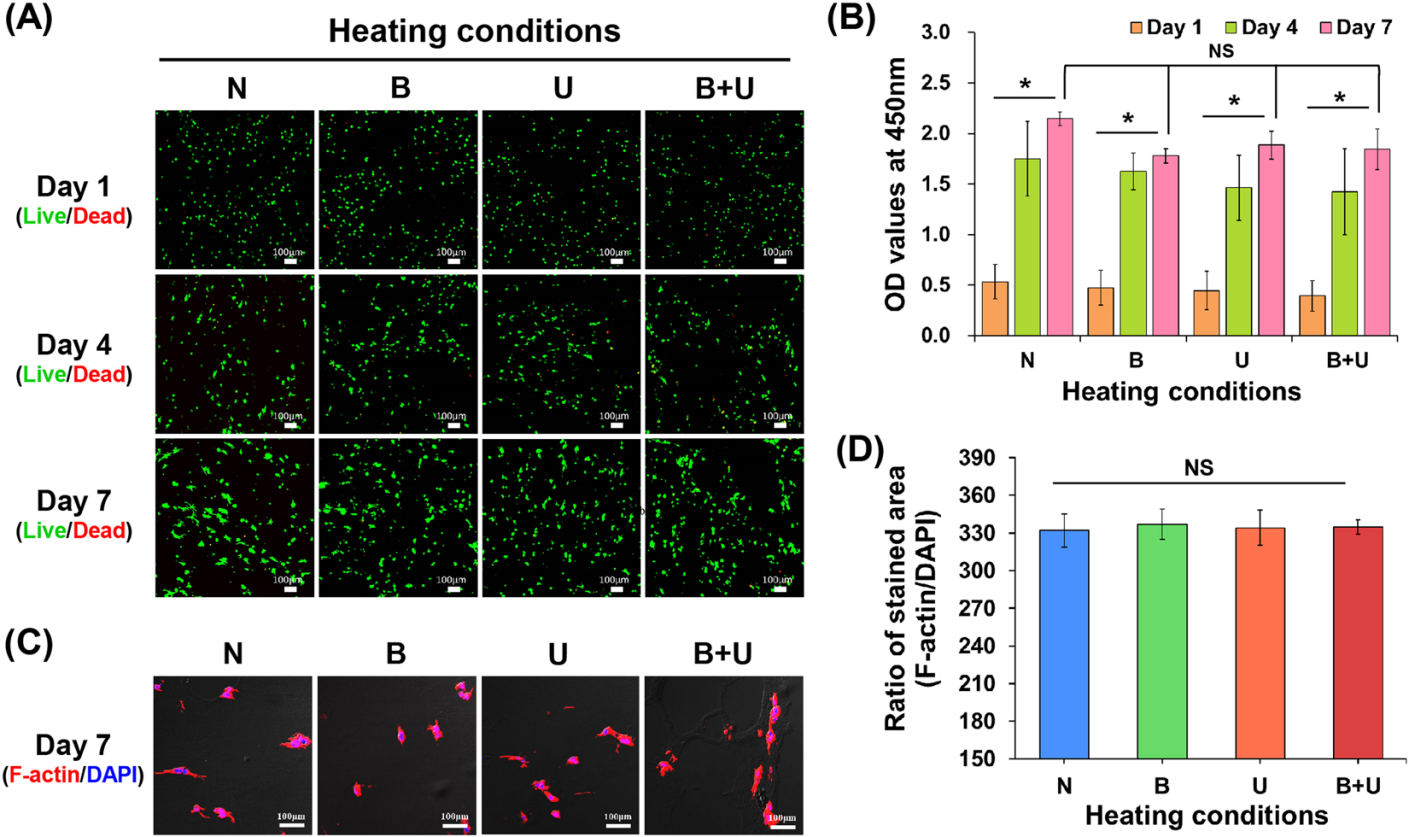
Analysis of cellular activities. (**A**) Live/dead assay results of NIH3T3 cells under different heating conditions. (scale bar: 100 μm) (**B**) Cell proliferation tests via CCK-8 assay. (*p < 0.001, NS = no significant difference) (**C**) F-actin (red)/DAPI (blue) images indicated cell spreading on day 7. (scale bar: 100 μm) (**D**) Analysis of the ratio of the stained area under different heating conditions.

With respect to printability, in this study, heating modules have been shown to yield superior outcomes; however, it is not clear how the thermal energy generated from heating modules affects cells printed with bioink. Figure 7(A) shows the cell viability results measured by live/dead assays. The printed cells under both non-heating (N) and heating conditions (B, U, and B + U) proliferated well for 7 days, without any damage. Figure 7(B) shows the proliferation results represented by OD (optical density) values after culture for 7 days (*p* < 0.001). During the culture period, the differences in OD values between all heating conditions were not significant. On day 7, the cells spread efficiently within the printed bioinks (Fig. 7(C)), and the ratio of the area stained by DAPI (indicating cell nuclei) and F-actin (indicating the cell cytoskeleton), used as a measure of the stability of the printed cells, did not differ significantly among heating conditions (Fig. 7(D)). Based on these results, we confirmed that the heating conditions for 3D cell printing did not negatively affect cellular activities, such as cell adhesion and growth.

### Stacking efficacy of the heating system

**Figure 8:**
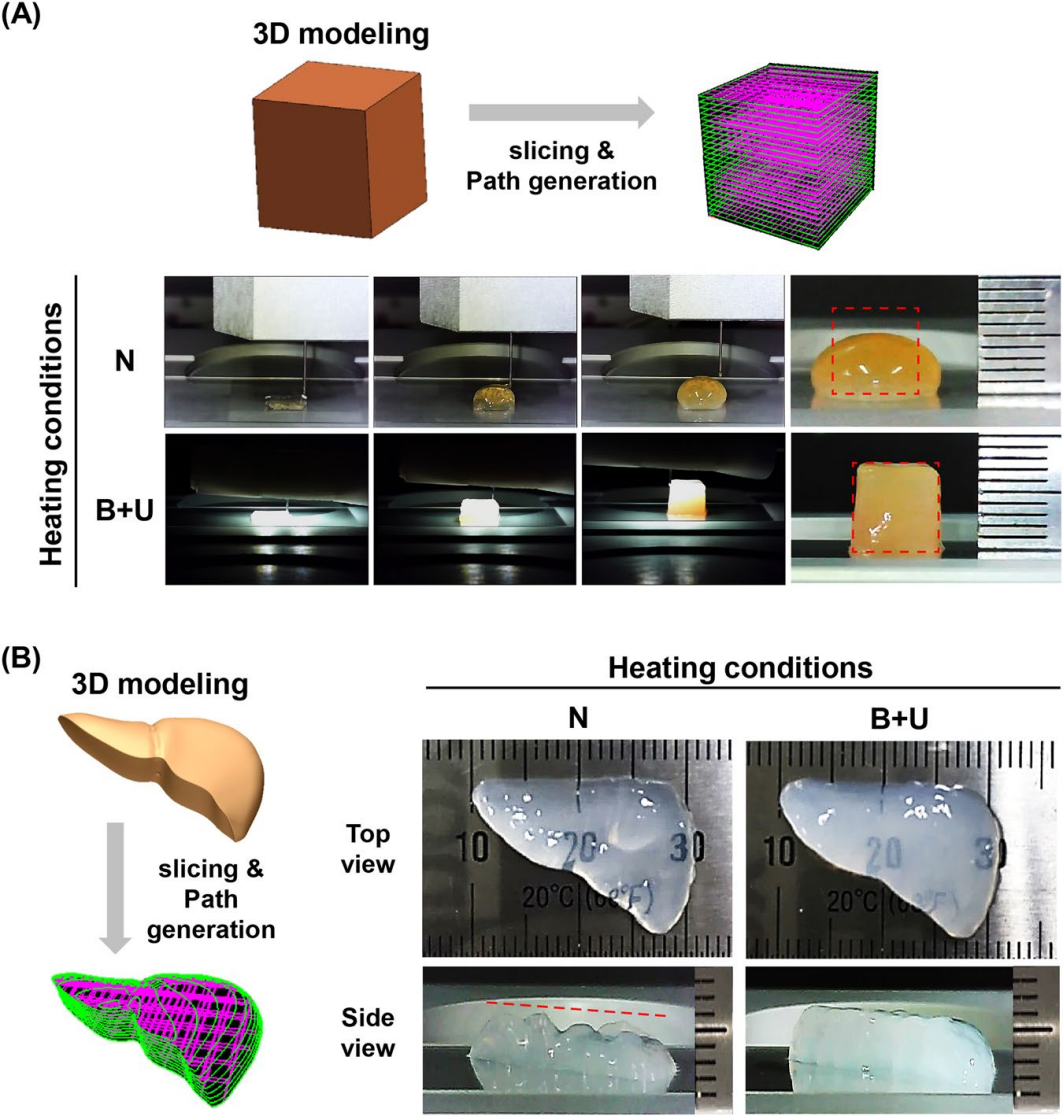
3D stacking results. (**A**) Observation of stacking efficacy of the heating system using rectangular-shaped constructs. Red-dotted square line indicates the predesignated shape. (**B**) 3D modelling of a liver-shaped construct and generation of printing code and 3D printing results of liver-shaped constructs under non-heating (N) and heating (B + U) conditions.

To confirm the effects of heating systems on printing quality, the stacking efficacy of the heating system was evaluated by building 3D constructs of 7.5 mm in height (25-layer stacking) without (N) and with a heating system (B + U) (videos S1 and S2). As shown in Fig. 8(A), when heating was not applied, the collapse of the structure occurred while stacking, but when heating was applied, the structure was well-stacked, while maintaining the predesignated shape. Furthermore, to demonstrate the 3D freeform fabrication, we printed mini-organ-shaped structures (videos S3 and S4). We designed a 3D liver model, and the printing code was generated by CAD/CAM (Fig. 8(B)). The printing quality under heating conditions (B + U) was superior to that under non-heating conditions (N). These findings confirmed that the heating conditions improved printing precision by providing a stackable environment to induce gelation.

## Discussion

In terms of cell printing, the characteristics of bioink and the printing process using bioink are very important factors. Many studies have examined biocompatible and printable bioinks to fabricate artificial tissues or organs. In particular, extracellular matrix-based bioink has been actively examined, with cell-friendly bioinks being essential for tissue and organ regeneration. In this study, we used dECM bioinks extracted from native skin tissue by decellularization and solubilization processes. Our results confirmed that the decellularization process for dECM bioinks was successful. The removal of DNA known to cause immune responses *in-vivo* is necessary for the use of allogenic and xenogeneic materials isolated from animal tissues or organs^6, 40^. Conversely, the retention of ECM components such as GAGs and elastin in the decellularization process is required to mimic the tissue-like environment. It could provide crucial biochemical signals for cellular activities, such as cell adhesion, proliferation, and differentiation^12, 40^. Despite these advantages, during the dECM production process it may be difficult to stabilize the yield of dECM having uniform properties (physical and biochemical) owing to inherent differences in raw materials.

The fluidic properties of bioinks are critical to print 3D cell structures^22^. Recently, some studies have evaluated the relationship between the rheological properties and the printability of bioinks^20, 22, 28^. In particular, the concentration of the bioink could significantly affect not only the microenvironment influencing cellular activities, but also printability (e.g. shape fidelity). For these reasons, the selection of a proper bioink is essential for successful artificial tissue/organ printing. The cell viability and printability of a bioink also have a special relationship. Accordingly, we confirmed the appropriate bioink concentration needed to produce a suitable cellular environment associated with cell viability. Based on comprehensive analyses, we confirmed that there was a tradeoff between cell viability and printability; bioink concentrations promoting cell viability did not show sufficient printability.

Generally, layer-by-layer stacking processes are widely applied in extrusion-based 3D cell printing systems^3, 15, 17, 20, 22, 24, 25, 28, 36^. The heights of 3D constructs could not be accurately estimated because the integration of each layer in the *z*-direction should consider the extent of bioink collapse owing to its viscoelastic features. This feature could ultimately lead to printing failure. Without the simultaneous gelation of bioinks, the collapse phenomenon was expected owing to the liquid-like properties of the bioink and the effect of gravity^36^. Therefore, precise 3D stacking of the bioink is important to fabricate the predesignated artificial tissue/organ constructs. We established a 3D cell printing system equipped with heating modules, which substantially improved the printability of bioink at a concentration appropriate for cell survival compared to the printability for the system without heating. Applying the bottom heating module (case B), the thermal energy transferred to the 3D printed construct was gradually reduced in the *z*-direction, as the newly deposited bioink was stacked up. In contrast, heat transfer by the upper heating unit (case U) at a constant distance (between the 3D printed construct and the heating unit) resulted in the formation of a uniform construct because the consistent thermal energy helped to induce simultaneous gelation at each final layer during printing. In cell printing with heating, the gelation efficacy for each condition (U, B, or B + U) showed meaningful differences in a compression test of the printed structures (Fig. 6(D)). Although the saturated temperature and G′ values were higher in the B condition than in the U condition, the compressive modulus of the U condition was significantly greater than that of the B condition. It could be concluded that thermal energy generated by U, which is located at a certain distance from the end of the nozzle, was more effectively applied to the bioink during printing compared with that generated by B. Moreover, the B + U heating condition included both characteristics (B and U), providing a proper environment for the thermal-sensitive gelation of dECM bioinks. This 3D cell printing system had the advantage of allowing the precise generation of cell-laden constructs by inducing simultaneous gelation of the printed bioinks and was a safe method with respect to the viability of printed living cells. Therefore, we conclude that this method is a robust tool for obtaining life-sized tissue/organ analogues.

Future studies are needed to further elucidate the biological characteristics (e.g. cell differentiation and tissue formation) in order to evaluate the maturation of target tissues in 3D cell-laden constructs fabricated by this heating system.

## Methods

### Preparation of dECM bioinks

Native skin tissues (corresponding to food waste) obtained from a Korean domestic pig, which was slaughtered for edible purposes, were collected and washed. The skin tissues were decellularized as previously described^41^. Briefly, the tissues were chopped, treated with enzyme and nonionic detergent, and then washed with phosphate-buffered saline (PBS; Gibco, Grand Island, NY, USA) for 24 h to remove cellular components. The decellularized skin samples were lyophilized for 24 h and the resulting powder was solubilized in an acidic pepsin solution for 3 days. To develop a printable bioink, the pH level of the solubilized samples was adjusted to 7.4 to induce gelation. To confirm the properties of the bioink, the residual DNA was measured using a commercial kit (G-spin Total DNA Extraction Kit; iNtRON Biotechnology, Seongnam, Republic of Korea), and the remaining dECM components including collagen (Sircol, Biocolor, Offenbach, Germany), glycosaminoglycan (GAG; Blyscan, Biocolor), and elastin (Fastin, Biocolor) were assessed by using each specific assay kit. All contents were normalized to the basis of native skin. The vibrational analysis was performed by Fourier transform infrared (FTIR) spectroscopy (Nicolet 6700; Thermo Scientific, Waltham, MA, USA). A small amount of the samples was mixed with potassium bromide (KBr) and pressed into a pellet. FTIR spectra were recorded in a spectral range of 4000–400 cm^−1^.

### Characterization of physical properties

The compressive mechanical properties of various concentrations of the gelled bioink (1.5%, 2.0%, and 2.5% weight per volume (w/v)) were examined using an Instron 3343 Mechanical Test System (Instron, Norwood, MA, USA). The gelled bioinks (10 mm in diameter and 6 mm in height) were placed on the centre of the system and compressed at a velocity of 1 mm/min by applying a uniaxial compression force. The compressive modulus was calculated from the linear slope in the initial portion of the stress-strain curve.

The rheological properties were measured using a controlled shear stress rheometer (Kinexus Pro + ; Malvern Instruments, Malvern, UK) and a parallel-plate geometry (20-mm flat plate). All measurements were performed with a 1-mm gap width at 25 °C. The shear viscosities according to different bioink concentrations were assessed using shear sweep tests (0.1 to 100 s^−1^). Additionally, temperature sweep oscillatory tests were performed with a single frequency (1 Hz) under 2% strain. A temperature ramp of 5 °C/min was applied from 15 °C to 40 °C.

### Scanning electron microscopy (SEM) analysis

All freeze-dried samples were coated with platinum for measurements of pore morphology using an S-4700 SEM (Hitachi, Tokyo, Japan) at an accelerating voltage of 15 kV. All SEM images were analysed to determine the mean pore sizes using ImageJ. The mean pore sizes were calculated from six randomly selected areas in the SEM images (n = 3).

### Printing of two-dimensional (2D) patterning

To determine printability, straight lines measuring 8 mm in length were printed for various bioink concentrations and printing parameters, such as pneumatic pressure (40, 60, and 80 kPa) and feed rate (50, 125, and 200 mm/min). For the analysis of printability, the ratio (D_N_/D_P_) was calculated by analysing optical microscope images using ImageJ software.

### Printing of 3D constructs

The 3D printability and integrity were assessed by building square-shaped constructs. Under non-heating conditions, a variety of bioinks were repeatedly stacked in the *z*-direction, and the maximum number of stacking layers was assessed. To identify the effects of heating modules on the 3D cell printing system, the heating temperatures of all heating conditions were measured using thermocouples. A commercial heating plate was used for bottom heating, and commercial LED lamps were used for upper heating. Under the heating conditions reached at each saturation temperature, a total of 15 layers were stacked in a hollow-type square with measurements of 10 × 10 mm in each dimension.

### Cell culture and cell viability analysis

Mouse fibroblasts (NIH3T3) were cultured in Dulbecco’s modified Eagle’s medium (Gibco) supplemented with 10% foetal bovine serum (Gibco) and 1% penicillin/streptomycin (Gibco) at 37 °C in a humidified atmosphere with 5% CO_2_. The medium was changed every 2 days.

Cell proliferation assays were performed using a Cell Counting Kit-8 (CCK-8; Dojindo Laboratories, Kumamoto, Japan) for 7 days. Briefly, the CCK-8 solution and serum-free Dulbecco’s modified Eagle’s medium were combined at a ratio of 1:10 and then added to each sample. After incubation for 3 h, the mixed solution was extracted, and the optical density (OD) values at 450 nm were measured using a microplate reader (Epoch; BioTek, Winooski, VT, USA).

For live/dead cell viability assays, samples were stained using live/dead staining solution containing 2 μM calcein AM and 4 μM EthD-1. The stained samples were observed using a confocal microscope (FV1200; Olympus, Tokyo, Japan).

### Statistical analysis

All data are expressed as means ± standard deviation. Statistical significance was determined by one-way analysis of variance (ANOVA) using MINITAB software (Minitab Inc., State College, PA, USA). Differences with *p-*values of less than 0.05 were considered significant. All experiments were performed in triplicate for statistical analyses.

## Electronic supplementary material

3D stacking results of rectangular-shaped constructs via non-heating system.

3D stacking results of rectangular-shaped constructs via heating system.

3D stacking results of liver-shaped constructs via non-heating system.

3D stacking results of liver-shaped constructs via heating system.

Supplementary Information
