# Supplementary material for: Precise stacking of decellularized extracellular matrix based 3D cell-laden constructs by a 3D cell printing system equipped with heating modules
Source: Sci Rep. 2017 Aug 17;7:8624. doi: 10.1038/s41598-017-09201-5 (PMC5561246; doi:10.1038/s41598-017-09201-5)
Supplement: Supplementary file 5 — Supplementary Information [file 41598_2017_9201_MOESM5_ESM.pdf]

Precise stacking of decellularized extracellular matrix based 3D cell-laden constructs by a 3D cell printing system equipped with heating modules

Geunseon Ahn, Kyung-Hyun Min, Changhwan Kim, Jeong-Seok Lee, Donggu Kang, Joo-Yun Won, Dong-Woo Cho, Jun-Young Kim, Songwan Jin, Won-Soo Yun and Jin-Hyung Shim

Supplementary Information

Supplementary videos

Video S1. 3D stacking results of rectangular-shaped constructs via non-heating system.

Video S2. 3D stacking results of rectangular-shaped constructs via heating system.

Video S3. 3D stacking results of liver-shaped constructs via non-heating system.

Video S4. 3D stacking results of liver-shaped constructs via heating system.
